# Releasing Three Orphaned White Rhinoceroses (*Ceratotherium simum*) to the Game Reserve in South Africa. Rehabilitation, Translocation and Post-Release Observations

**DOI:** 10.3390/ani10122224

**Published:** 2020-11-27

**Authors:** Katarzyna Miazga, Johan Joubert, Megan Sinclair, Anna Cywińska

**Affiliations:** 1 Department of Pathology and Veterinary Diagnostics, Institute of Veterinary Medicine, Warsaw University of Life Sciences (WULS-SGGW), Nowoursynowska 159c, 02-787 Warsaw, Poland; anna_cywinska@sggw.edu.pl; 2 Shamwari Private Game Reserve, SPGR, Eastern Cape, Paterson 6130, South Africa; johan.joubert@shamwari.com (J.J.); vet.nurse@shamwari.com (M.S.)

**Keywords:** white rhinoceros, wildlife rehabilitation, conservation

## Abstract

**Simple Summary:**

Every year, thousands of African animals die at the hands of poachers. One of the most famous species killed by poachers is the rhinoceros, particularly the white rhinoceros (*Ceratotherium simum).* Out of many methods of dealing with this situation, the rehabilitation of wounded and/or orphaned animals with the intent to successfully release them back into the wild is becoming more encountered. This study presents the history of successful release of three orphaned white rhino females; rehabilitated for 15 months in Wildlife Rehabilitation Centre in a private game reserve located in Eastern Cape in South Africa. The animals differed in age and size: female A was three years old, female B was one year old, and the youngest one was three months old on arrival. The procedure turned out to be sufficient to keep their natural habits and since they were released, they have been living in the wild successfully.

**Abstract:**

White rhinoceros (*Ceratotherium simum)* is one of the most famous victims of poachers in Africa. One of the methods for dealing with decreasing rhino numbers is rehabilitating wounded and/or orphaned animals to successfully release them back into the wild. The status of rescued animal differs among individuals, but general procedures must be established and constantly improved. This study presents the history of successful release of three orphaned white rhino females; rehabilitated for 15 months in Wildlife Rehabilitation Centre in a private game reserve in South Africa. Female A was three years old, female B was one year old and the youngest female was three months old on arrival. The animals were rehabilitated together despite the differences in their age and size, with particular attention paid to keeping them as wild as possible. After being weaned and becoming old enough to go back to the wild, they were released at a distance from the rehabilitation centre, which required immobilization and translocation. Since the rhinos were released, they have been successfully living in the wild. All procedures used in this study proved to be sufficient for preparing the animals for life in the wild and can be recommended for other centres.

## 1. Introduction 

White rhino (*Ceratotherium simum*) is one of the most iconic African species. It is one out of two existing species, together with black rhino (*Diceros bicornis*), that lives in the wild on this continent. As one of the megaherbivores, together with hippo, their role in the ecosystem is crucial in maintaining a balance. By consuming large amounts of plants, more than 50 kg per day, they help in shaping the landscape and mark indirect effects on their habitat by altering fire regime by shortening grasses and creating biologically induced barriers to the spread of fire [1]. About 20 kg of droppings per day fertilize the soil and help spread the seeds of many plants. They also help in creating natural waterholes by rolling in mud puddles. Being hosts to various species of ecto- and endoparasites, rhinos are an important part of their life cycles [2]. Furthermore, rhinos play a huge part in the tourism industry as a part of the “Big Five”. Their numbers have risen significantly in the last 100 years, from fewer than 100 individuals in the early 1900s to roughly 20,000 today [3]. The vast majority of the wild population, almost 91%, occurs in South Africa and Swaziland [4]. Current status, according to IUCN Red List of Threatened Species is “near threatened” [5]. Still the vision of becoming extinct does not seem to be unreal and far away. The reason for that is the scale of poaching which has been alarmingly growing since 2007 [3]. Asian countries are the main market for rhino horn trade, including Vietnam and China, where according to traditional medicine, rhinoceros’s horns are attributed to healing and even magical properties including dispelling the heat from the body, detoxifying blood, treating cancer or hangover and others [6]. The horn is also seen as an excellent fine art curving material used to make cups, thumb rings as well as other functional or ornamental items [7]. In 2018, China revoked its 1993 rhino horn trade ban and reopened its domestic trade under two conditions, namely that horns must be sourced sustainably, and the use of the horns must be limited to traditional Chinese medicine, medical research and preservation of antique cultural artifacts and educational materials [8]. Over the last several years, rhino horn pieces are portrayed in the Chinese media as an investment opportunity whose value is tied more to the rarity of the raw materials rather than artistic nature of the item. Illegal rhino horn trade also continues in other forms of trade such as online black markets and in other countries [7].

In 2007, 62 cases of poached rhinos have been reported in Africa, while in 2015 the number increased to 1349 individuals [3]. These are the cases when the carcasses were found and recognized as the result of poaching, but many remains may have not been discovered. The South African Department of Environmental Affairs announced that 318 rhinos have been poached in the first six months of 2019, with the majority of white rhinos which are a much easier target for poachers as they graze in the open areas and are less aggressive than black rhinos [9]. Moreover, their population is much larger than black rhinos, making them more available for poachers. Taking these statistics into account, together with the number of animals dying for other reasons (e.g., age, diseases) and the breeding cycle of this species, reaching sexual maturity at the age of 5–7 years, pregnancy lasting 16 months and only one calf at a time, it might turn out that every year the number of animals lost is greater than those that are born [10]. Between 1991–2010 the annual growth of the white rhinoceros population in South Africa was on average +6.6% per year [11]. Since 2008, population estimates have indicated that annual growth rates for white rhino was fluctuating around zero and beginning from 2013 data have suggested that the growth rate may be even negative or at least fast approaching negative values [12]. This is why every individual of the white rhinoceros is extremely valuable for the survival of the population. Considering the growing threat of losing this species many efforts are being done to apply valuable measures to counter poaching and deal with its effects. The research conducted in Kruger National Park indicates that birth rates may decline with increased density and decreased rainfall [13]. That is why strategic rhino removals are considered as one of the relevant ways of protecting and controlling the population [12,13]. The main purpose of the Biodiversity Management Plan between 2015–2020 is to ensure a long-term survival in the wild of the white rhinoceros [11]. The conservation target is to achieve a metapopulation of at least 20,400 animals in South Africa by 2020. The key essential components listed by the authors are biological management, protection, monitoring, permitting and stock control, effective communication and collaboration, hunting and sustainability. Carefully planned actions and wide cooperation at different levels gives hope for satisfactory results. Furthermore, controlling and knowing the population composition allows to ensure genetic diversity and avoid inbreeding. 

An increasing number of mutilated and orphaned animals resulted in creating rehabilitation centers that help the victims of poachers. The most common aims of rehabilitation are animal welfare and conservation [14]. The process that animals go through in this type of centers can be divided into three main stages—Rescue, rehabilitation and release [15]. Associated research in each phase give the opportunity to improve the process and make it safer and more effective (4 Rs—rescue, rehabilitation, release, research) as it has been reported in birds, mammals, reptiles, and amphibians (469 species in total). Animals are rescued if they are injured by human or other animals or considered in human-related danger. If they need additional care, the rehabilitation or recovery phase is provided before releasing them to the wild. The procedures during each stage are aimed on helping animals to live normally in the wild in future. The definition of normal life may not be obvious. In case of white rhinos, it can be stated that normal means showing characteristics of white rhino found in the wild which are considered to be normal and comparing them to the behaviors of the rhino which were in the rehabilitation centre. At any stage of the process, many risks and unexpected factors may occur. Although each case is specific in age, the general health status and the history, the general rules for certain species must be established and kept, but also constantly improved by every observation that maybe helpful. General procedures and principles are defined as the conditions of keeping this species in captivity, such as construction of the enclosure, diet of both bottle-fed and solid food animals, methods of monitoring animals’ condition, protocols in case of veterinary interventions, making decisions regarding release, conducting the release and in some cases translocation and post-release observations. Some experiences from rehabilitation centers must be kept in secret to protect the animals from poachers but some details, if published, can be extremely helpful for other centers. 

One of the newest facilities is the Wildlife Rehabilitation Centre (WRC) opened in 2019 in one of the reserves located on the Eastern Cape in South Africa. The first major success of the newly opened center was raising and releasing three orphaned female white rhinoceros. This study presents the critical steps of this process but also details that can be helpful in further procedures of 4 Rs in white rhinos.

## 2. Materials and Methods 

### 2.1. The Origin of the Animals

Three white rhino females were brought to the Rehab Centre in May 2018. Two of them, subadults aged about 3 years (female A) and 1 year (female B), came from a reserve in Mpumalanga province. Both of them were orphans of poaching. The third female (C) came from a reserve located in the Eastern Cape and was 3 months old at that time. Her mother was killed by another rhino. All rhinos came from reserves where details on dates of birth where documented, thus their exact age was known. Female A was old enough to be released on arrival however, it was decided to wait for the other two to be old enough and release them all together. Adult females frequently graze together in groups and bonds between them may be quite strong and they will co-operate to defend themselves against predators [16,17,18,19].We hoped that company of other individuals of the same species will make the rehabilitation process smoother and that they will stay together after the release, which would make it easier for them to accommodate to new conditions. Given relatively gregarious nature of white rhino females keeping them in groups is desirable. In case of males, keeping several individuals in one enclosure is not recommended due to the possibility of fights between them [18]. 

The main goal of the project was to raise three white rhino orphans, prepare them to live a human-independent life in the bush, safely translocate and release them. It was expected that the rhinos will be able to survive without human support, understood as fend themselves, finding food, water, and shelter on their own, not trying to make contact with people and breed in future. All these criteria are monitored by observations based on radio collar location.

### 2.2. Rehabilitation Care 

In the beginning all animals were kept in the enclosures in the area of the reserves Wildlife Department as the new WRC was still under construction. Females A and B were already able to consume solid food, so they were fed with grass growing in their enclosure and lucerne hay given once a day. Both of them were scared of people and rather avoided contact with them. The youngest female (C) was still dependent on milk when she arrived. She was fed four times a day with the feeding mixture consisting of foal replacement milk powder (Denkavit Foal Milk, Denkavit Netherlands BV, Voorthuizen, The Netherlands) with addition of electrolytes (Pro-Lyte with Glutmaine, Kyron Laboratories (Pty) Ltd., Johannesburg, South Africa) and intestinal probiotics (Protexin Soluble, Kyron Laboratories (Pty) Ltd.). Due to the difference in age and size, two older cows were kept separately from the little one, however all animals had eye contact through the fence separating their enclosures. To provide the youngest female with company, an adult sheep was introduced to her one month after her arrival to the Rehab Centre. A sheep was chosen because this species is characterized by a very strong herd instinct and calmness, and the size is relatively safe to manipulate when needed. Previously, this species was used successfully as a companion for an orphaned baby elephant. Another rhino would have been preferable instead of sheep, however there are many additional issues could have arisen. It would not be ethically accepted to take a wild rhino and place it in captivity for the purpose of raising an orphaned calf. Moreover, the wild rhino could injure or reject the orphan. There were no other orphans available at that time and the two older females were too big and could hurt the smaller one. Thus, the sheep has been selected as the best companion in such a situation. After being weaned and old enough, the youngest female joined the other two orphans. As rhinos spend 49% over a 24 h period feeding the size of the enclosure and vegetation are considered one of the most significant factors when it comes to stimulating natural activity cycles in captive environments. [18]. After opening the new Wildlife Rehabilitation Centre, all three animals were moved to a new 2 acres (0.8 ha) enclosure. The area and the growing vegetation reflected the conditions prevailing where the animals were planned to be released as accurately as possible, because the center was built inside the reserve. It is impossible to fit all types of vegetation occurring on the reserve in one enclosure, however, natural grass in the enclosure consisted of *Themeda* and *Conydon* and both of them are preferred in the wild rhinos’ diet [20]. In addition, animals had vegetation at their disposal, including other grass species and bushes, growing freely in the enclosure. For the browsing rhinoceros, a 1:1 mixture of grass hay and and lucerne hay has been recommended in order to mimic the nutrient composition of the natural diet, therefore extra food was supplied to them every day from a car driving into the enclosure and consisted of lucerne, hay with small addition of teff [21]. Forage was spread in different spots in the enclosure encourage animals to search for food and as a part of enrichment. Access to fresh water was provided permanently in a large trough with constant water circulation. Throughout the stay in WRC the animals were looked after by qualified carers. Their duty, apart from feeding, was to observe animals and monitor their health. Aspects such as body weight, appetite, feacal consistency, and changes in behavior, e.g., apathy or separating from the others, was taken into account. To evaluate animals’ condition the system for scoring body condition of white rhinos was used, based on the criteria described earlier, involving visual evaluation of reduction of fat deposits and muscle mass [22,23] (Table 1).

In case of any disturbing symptoms the veterinary team was informed and then it was up to them to decide on further management or treatment, adequately to current situation. At this stage, contact with animals has been reduced to a minimum to restraint human habituation, and prevent them from getting too used to human presence and sight, which may have resulted in association people with food. Hands-off rehabilitated rhinos are more alert and more social in comparison to hands-on animals [24]. This applied to the youngest female in particular, due to the fact she was bottle-fed for some time after she came to the reserve. Released animals lacking adequate behavior towards humans may become easier target to poachers [25].

In addition, other orphaned animals’ representatives of two antelope species, a blesbuck (*Damaliscus pygargus phillipsi*) and impala (*Aepyceros melampus*), were placed in their enclosure, so that both rhinos and antilopes could get used to the presence of other animals and learn how to coexist with them, e.g., gest used to the sight of different animal species and respond to their warning signals.

**Figure 1:**
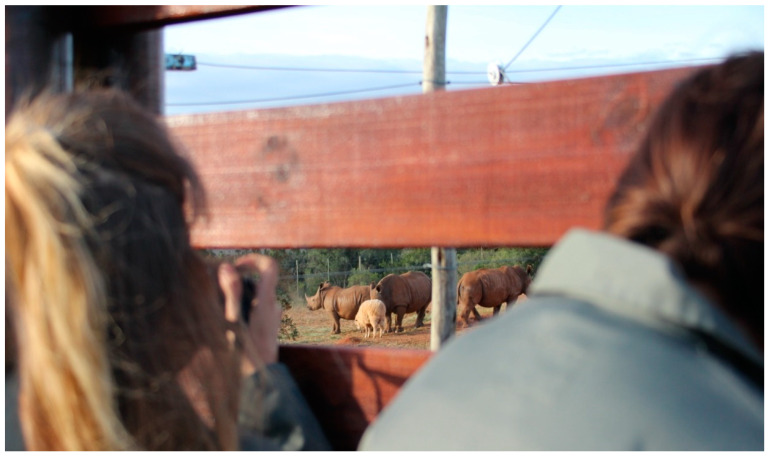
Observing rhinos on their enclosure in WRC through the small gaps. The gaps are narrow enough to hide humans and exclude the eye contact with visitors.

As WRC’s goal, beyond wildlife rehabilitation, is education, guests can visit the facility and learn about African wildlife conservation. The visitors include both tourists visiting the reserve and students from nearby schools and locals. It should be emphasized that no visitor–animal contact nor animal petting is allowed. Special covers are placed on the fence around all enclosures, so that the animals staying in the WRC do not have eye contact with visiting guests. Through the gaps located on the observation platforms, the animals can only see fragments of the faces of the observers (Figure 1). This allows visitors to observe different species without disturbing them, unlike in zoos where visitors can view animals from different angles [25]. Sight is not the strongest sense in rhinos, as opposed to olfactory and hearing abilities, which are both very acute. That is why such fence construction does not really apply for this species as an awareness of human presence, but it still provides more intimacy for the animals

### 2.3. Decision Regarding Releasing

White rhino calves are weaned at 18 months and generally stay with their mothers until 2–2.5 years, when they leave and join up with other rhinos. They become sexually mature at 5 years of age and are big enough to defend themselves from other rhinos [16,17,26]. Based on this knowledge, when the animals reached the right age (4.5 and 2.5 years for females A and B and 1.5 years for female C) and size to live on their own, the decision was made to reintroduce them back to the wild. Release decision was based on the observations confirming that all three animals were ready to live in the wild. They were old enough, none of them required bottle-feeding, they were grazing and natural behaviors seen in wild animals such as mud wallowing and body scratching were observed [24,26]. Moreover, the females showed bond, manifested by spending time together in the enclosure both during day and night as well as gathering together in case of potential danger. To ensure the progress of animals in the wild and care for their safety, it was decided to release them in the private game reserve. The reserve covers the area of over 25,000 hectares and has a stable white rhino population, that grew to a maximum capacity, despite isolated cases of poaching and the fact that many individuals have been translocated to other reserves over the past ten years. Poaching is still a real risk but another great advantage of the facility is the presence of specially trained Anti-poaching Unit (APU), with dedicated members who patrol the reserve and guard animals living in it. The release sight was selected where there was substantial natural grass, water, and the area was not densely populated by other white rhino. To provide full reintroducing to nature and to meet the animals and human safety conditions, the decision was made to release animals at a distance from WRC, which required immobilization and translocation. The whole action required many people and heavy equipment. The team consisted of the Wildlife Department members, including veterinarians and vet nurse, APU troopers, WRC employees and volunteers. Everyone was thoroughly informed about the translocation process and prepared for possible complications. 

### 2.4. Immobilization and Translocation 

The reserve holds a standing permit for rhino immobilization issued by The Department of Environmental Affairs. As animals were to be released on the same reserve, no translocation permit was necessary in this case. To avoid the risk of high temperatures during the day, as it was November, the whole procedure began in the early morning. The older cows (A and B) were darted with a mixture of 3.8 mg (0.38 mL) etorphine (Captivon 98, Wildlife Pharmaceuticals (PTY) LTD) and 35 mg (0.7 mL) azaperone (Azaperone, V-Tech). For the younger rhino (C), the following mixture was used: 3mg (0.3 mL) of etorphine and 25 mg (0.5 mL) of azaperone. Then, 2cc darts with 13 GA needles with wire barbs (Pneu-Dart Inc.) where filled up with the drug mixtures. The rhinos were darted individually by the vet from a vehicle in their enclosure. The placement of the dart was either the scapula or thigh region. Animals were recumbent after 10–15 min from darting. Two rhinos fell down in the lateral position while the third one settled down in sternal recumbency. Right after approaching each animal, blindfolds and earplugs were applied to minimize the number of stimuli and darts were removed. Every animal was constantly monitored by delegated members. Number of breaths per minute, color of mucous membranes, and depth of anesthesia were noted every 3–5 min. The number of breaths per minute was counted by observing the movements of the chest and the sounds made during exhalation. Simultaneously, the depth of anesthesia, together with ears movement, was controlled in this way. The color and capillary refill time of the conjunctival mucous membranes of the eye allowed to monitor cardiac function and indirectly the blood pressure. Before loading, every individual was equipped with a telemetry transmitter placed on the front limb, to track animals in the future and monitor their movements. Specific and unique ear notches were made on every individual for future identification on the reserve from a distance. The biological material collected from their ears in this way, was appropriately secured and then introduced into the rhino DNA data base where samples taken from the animal are archived and then can be used during the investigation and help find both the poacher and associate him with his principal [27]. 

**Figure 2:**
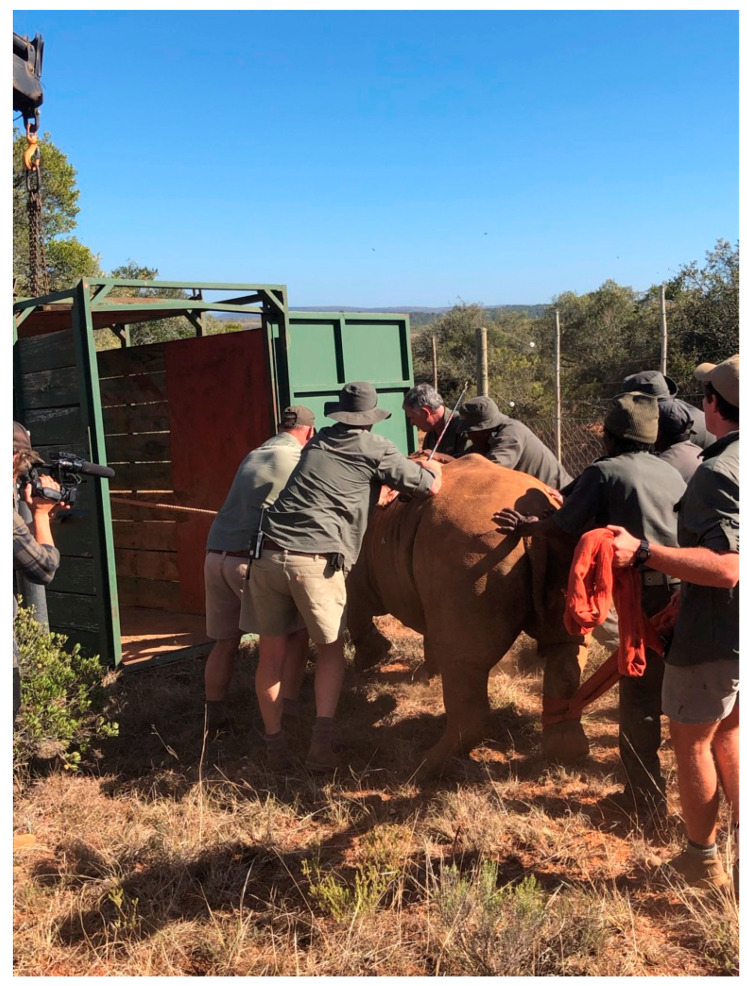
Walking the rhino into the crates. Animal is led to crates pulled by a rope tied around its head and pushed from the back at the same time.

Cow A and B were placed in separate crates by using the “walking” a rhino technique. Both were given 25 mg (0.5 mL) of butorphanol (Butorphanol, Kyron Prescriptions cc) intravenously. After 5 min animals were able to stand up but were unaware of what was going on around them at the same time. Using ropes tied to their head and legs, the rhino cows were led to their assigned crates, where the ropes were taken off but blindfolds and earplugs were left in (Figure 2). 

Next, the crates with animals inside where placed on the truck using a crane. Secure straps where used to keep crates as stable as possible. Rhino C was also given butorphanol, 25 mg (0.5 mL) intravenously but she did not manage to bring herself to a standing position. Thus, it was decided to put the animal on rubber stretcher, which was then pulled on to the truck. After removing the stretcher from under the animal together with blindfolds and earplugs, the etorphine antidote naltrexone (Trexonil, Wildlife Pharmaceuticals (PTY) Ltd., White River, South Africa) at a total dose 30 mg (0.6 mL) was administered intramuscularly. It took 10 min for the cow to stand up. After making sure that animals are breathing and both crates and truck door are secured, the convoy started the journey to the place planned for the release on the reserve. The whole journey took about 20 min. The rhinos were stable in the crates and their health was constantly monitored. 

### 2.5. The Release and Post-Release Observation

After reaching the destination crates and truck where placed next to each other with the exit facing the open area and the nearby waterhole in the bush. After removing all the blindfolds and earplugs, A and B were given 50 mg (1 mL) of naltrexone (Trexonil, Wildlife Pharmaceuticals (PTY) Ltd.) intramuscularly. Rhino C was already wide awake. Just before the animals were released, a few cubes of dried lucerne were scattered about 15 m from the trailers. When all animals where conscious and awake, all doors where open at the same time. Telemetry tracking collars placed on the animals while they were immobilized allowed for detailed observation.

The key points in the rehabilitation and release process are presented in Table 2.

## 3. Results

### 3.1. Rehabilitation Phase

After moving all rhino cows into new Wildlife Rehabilitation Centre, the care of their natural instincts, including human habituation were particularly important. The animal’s relationship with food-giver change over time. In the early stages, the animals may be passive recipients of the food handouts, but eventually they tend to become active solicitors of food from humans [28]. Females A and B were quite wild, scared of people, and rather avoided contact during the entire rehabilitation phase. Everyday food delivery did not change their relation to people. After being weaned, female C has learnt how to graze. 

Basing on our observation and experience with similar situations in the past, we assumed that a bond between the animals was formed, as they were inseparable while grazing, sleeping and playing together. Prior to release, all animals reacted to the human presence and expressed alert behavior typical for wild rhinos, such as gathering, perking up their ears, sniffing, and monitoring the surroundings [26].

During the whole process all animals were in good condition and their index score was defined as “fair” or “good” according to the index score (Table 1). None of the animals required veterinary treatment during the rehabilitation phase. 

### 3.2. Translocation, Release and Observation

During immobilization, all measurements in females B and C were satisfying. An average of 7 breaths/min was recorded, which was the desired and appropriate value. During the ride, rhino A began to develop hyperthermia, manifesting as shallow and rapid breathing as well as moderate sweating with limited signs of waking up. Water was used to cool her down.

Lucerne was placed around the trailers to help lure the rhinos out and to ensure they were all able to eat normally. All three females walked out and started calling each other immediately. After about 10 min they began to eat with no signals indicating disturbed appetite or problems with food intake. After approximately 5 min they started walking towards the bushes. 

Suddenly, an unexpected situation occurred, nobody has ever anticipated such circumstances before. About 15 min post release, an adult male white rhino appeared from the nearby bushes and started walking towards the three females. We would expect wild animals to run away or observe the whole procedure from a safe distance as engines and people caused quite a stir and noise. 

**Figure 3:**
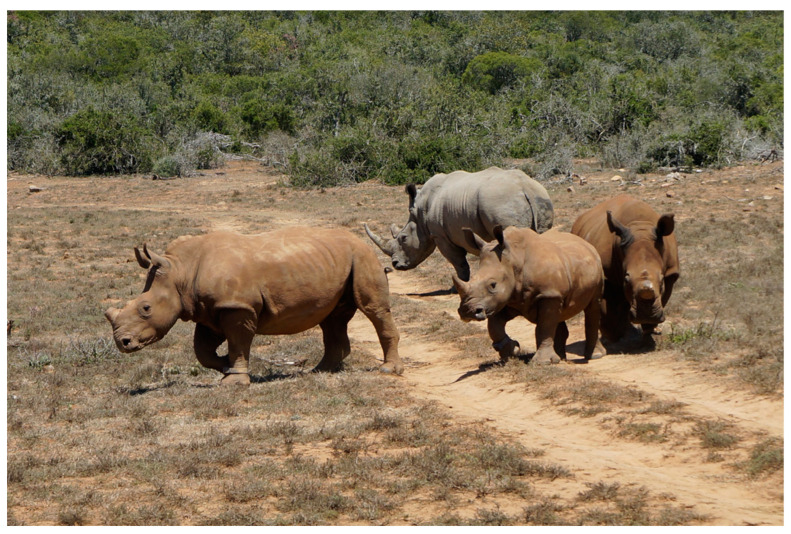
Three released females accompanied by a wild male 15 min post release.

As nothing could be predicted, the decision about no additional actions was made immediately. The worse scenario could be an attack of the male defending his territory. In this case, the females, despite being outnumbered, would not have much chance and would have to separate and run away to escape from potential danger. Fortunately, the situation resolved in the best possible way. After sniffing and getting to know each other, all four rhinos started grazing and finally moved away (Figure 3). 

Ever since the release, the animals have been regularly monitored by conservationists and APU members. Tracking the animals was possible thanks to telemetry tracking collars placed on the animals while there were immobilized. During this period of time nothing disturbing or indicating problems with adapting to the new situation was noticed. All three females are eating well and are able to find water by themselves.

About two weeks post release, it was noticed that the youngest female (C) separated from other individuals and leads a solitary life since then. Observations show that it did not affect her condition or health. She is being seen in the valley nearby the release spot while the other two are walking quite far around the reserve. Exact movement pathways and territory cannot be revealed due to safety precautions. Observation and control of the animals’ condition are constantly carried out, with special attention paid to health and reproduction. The attention is payed to the weight (body condition score) of the animals, especially checking if any ribs are visible. To estimate the body condition score int the wild, the same scale as in the WRC is used (Table 1). The dung is also monitored to make sure that they are eating well and drinking substantial amounts of water (Table 3).

## 4. Discussion and Conclusions 

At the moment it can be concluded that all animals have adapted well to the new living conditions in the wild. 

Wildlife began to suffer when the encroachment of urban areas and settlements started taking up their “truly wild” spaces. The only way one has been able to preserve these wild spaces is to place fences around these areas to contain the animals inside the fence and preserve these “truly wild” areas. Having a fence surrounding a reserve does not make the animals any less wild then the ones that live without fences. There are no interactions with these animals and breeding still takes place naturally, thus keeping the animals wild. Management of the area and animals within the fence needs to take place, as predator: prey ratios need to be monitored [29].

### 4.1. Human Habituation and Safety in the Reserve

In case of white rhinos, if they are not injured but only in human-related danger due to becoming orphaned, no special training is needed during rehabilitation phase. It is important to reduce human-animal contact prior to release. It is essential for animals to have a normal fear of humans and maintain adequate distances and the fear of humans is necessary to avoid interactions with poachers. Under wild conditions, white rhinos show fear towards humans, manifested by alert behaviours and alert response [24,26]. The care to keep their natural habits and avoidance of people seem enough for successful release to wild. Still, it cannot be forgotten that this species needs certain living conditions and appropriate facilities as well as trained and experienced team of people taking part in both rehabilitation phase as well as release and then monitoring of animals in the wild. The following problem is human and animal safety. The case of Thula Thula Rhino Orphanage from 2017, shows that such facilities can be an easy target for poachers [30]. The orphanage has been attacked by armed men during the night. Unfortunately, some animals were killed and their carers got injured.

### 4.2. Actions Taken to Protect Rhinos

Due to the poaching crisis in Africa, variety of actions are being taken to protect rhinos from human related risk. The presence of APUs in game reserves is now more encountered than it used to be in the past. Their duties include patrolling the reserve, inspecting fences and direct monitoring and guarding animals. The use of modern technologies, such as sirens or drones, also allows them to chase away animals from potentially dangerous areas that are easily accessible to poachers, such as fence lines and large open spaces [31]. Unfortunately, such activities prove to be insufficient and sometimes other steps need to be considered in order to protect rhinos. Recently, one of the most common action is shortening the horns of previously anesthetized animals. Dehorning aims to discourage poachers from shooting animals. However, the horn grows back at a rate of about 5 cm per year in adults and 6 cm in subadults [11]. Another idea was presented by a group of scientists, who has successfully created a rhino horn substitute, made of horse tail hair and silk [32]. A recent and innovative project is the embryotransfer of oocytes obtained in ovum-pick up, fertilized by intracystoplasmatic sperm injection, and developed to the blastocyst stage in-vitro [33]. 

A completely different approach to protect animals against poachers is the proposal to legalize rhino horn trade which has been banned since 1977. The proposed sources of the horn would be: horn derived from rhinos that die of natural causes, horn derived from living rhinos that are dehorned at intervals, horn derived from trophy hunted rhinos and from stockpiles currently held by state and private owners [34]. Accurate analyses and calculations have shown, however, that such an action has no chance to meet the demand on the Asian market. On the contrary, it can be increased even further. In addition, legalization will remove the stigma associated with the consumption of illegal products of animal origin and then counteract mandatory programs for changing human behaviors which seems to be the best solution against animal crime [35]. 

### 4.3. Releasing Rehabilitated Rhinos

Regardless the methods listed above, releasing the rehabilitated rhinos to the wild will be constantly provided. The procedure presented in this paper gives hope for the success of such ventures in the future. Studies have shown that about 18% of white rhinoceroses placed in bomas for three years are deemed maladapted, as indicated by the scoring system based on the appetite, fecal consistency/volume and behavior [36]. In our study, the mentioned scoring system was not directly applied, but overall behavior and health was constantly monitored and no disturbing symptoms, that could suggest any health issues were observed.

### 4.4. The Use of Veterinary Medicinal Products 

The decision regarding releasing included the use of standard method for immobilization with the mixture of etorphine and azaperone, most often used in this animal species [14,37]. Etorphine is an extremely potent and fast acting opioid, which makes it suitable for immobilizations of large animals. The list of its side effects includes respiratory depression, severe hypertension, altered thermoregulation, pure muscle relaxation and muscle tremors. That is why etorphine is administered together with a tranquilizer or sedative which act synergistically with etorphine and reduce muscular hypertonicity. Butorphanol, as a partial antagonist (kappa-agonist and miu-antagonist) has the ability to reverse the effect of most potent opioids, including etorphine and improves breathing at the same time. A study on the effect of administering butorphanol after immobilization of an animal with an etorphine-azaperone mixture confirmed a positive effect on the respiratory system by improving the ventilation in comparison to the control as indicated by lower P_a_ CO_2_ and an improvement in in P_a_ O_2_ [38]. It was also indicated that the addition of diphrenorphine did not offer any advantage over butorphanol alone [39]. Moreover, cardiovascular reactions in the form of tachycardia and an increase in blood pressure have been observed in rhinos immobilized with etorphine. The addition of azaperone to etorphine in the dart, allows a reduction in blood pressure and reduces muscle tremors which further reduces blood oxygen levels, while intravenous administration of butorphanol in recumbent animals causes the heart to slow down to normal values, most likely due to reduce the hypoxemia [40]. The study conducted in two groups of rhinoceroses, free-ranging and boma-held individuals, confirmed the effect of intravenously administered butorphanol on decreasing the muscle tremor intensity in both groups of animals [41]. 

Thus, in our study, butorphanol was used to walk the rhinos to crates on their feet instead of lifting totally unconscious animals by a crane and ameliorate their breathing. Similar method was recently described for giraffe translocation, however naltrexone instead of butorphanol was used. After immobilizing giraffes with etorphine and azaperone, once the individuals were recumbent, naltrexone was administered intravenously to antagonize the opioid-related side effects. Next, blindfolded and secured with ropes, animals were encouraged to stand up and guided into the chariot [42]. 

### 4.5. The Adventages of Translocation

Releasing rhinos without immobilization and translocation has been reported this year in the Hoedspruit Endangered Species Center [43]. Two rehabilitated rhino males were sent free by opening the gate of their enclosure. In the beginning they were following the car which was used to feed them and eventually they disappeared in the bush. In our study, it was decided to translocate the rhinos. If animals were released too close to the WRC, they could destroy the fence by trying to get to a place they know and recognize as safe. This, in turn, could pose a threat to other animals staying in the center. For this reason, it was decided to release the animals at a distance from their enclosure. Main challenges to animal welfare during transport of rhinos are dehydration, energy balance, skeletal muscle fatigue and stressed-induced immunomodulation. Such observations were made when transporting rhinos over distances of up to 1300 km [44]. In our case, the distance between WRC and releasing spot was much shorter, however, the animals’ health was constantly monitored during the drive. The constantly challenging pathology that can occur after immobilization and transport is capture myopathy, known also as stress myopathy or idiopatic muscle necrosis. This condition is quite well-known but still not well-understood and is characterized by severe muscle rhabdomyolysis, acute kidney failure and elevated body temperature. It occurs in land mammals but also in marine mammals such as dolphins and reptiles and birds. At the moment it seems that good planning of capture (e.g., time of the day, drugs mixtures, recumbency, translocation) is the best way of improving survival rates of wild animals [45,46].

Due to the drought in the region for a long time, it must have been considered that the animals would encounter difficulties in finding a waterhole. Studies conducted in the Kruger National Park have shown that white rhinos are much more sensitive to lack of water than black rhinos. During long periods without rain, researchers noticed an increase in mortality and a decrease in reproduction in this species in the local population [47]. Therefore, releasing the animals close to the water source and making sure that they could find it was extremely important. It seems that all animals in our study have accommodated to the new living conditions very well. All three animals are healthy with no indications of unusual or pathological behaviors, though our ability to make such assessments is constrained by available information based on observation.

The presented success gives hope that the released animals will breed in the future and thus they will contribute to increasing the number of their species in the wild. Taking into account a decrease in genetic diversity in this species, resulting, among other things, from the monogamous nature of females, incest cases and limited migration possibilities in a restricted reserve area, releasing three females originated from other reserves can significantly contribute to enrichment and, as a result, the strengthening of the local population of white rhinos [48].

## Figures and Tables

**Table 1:** System of scoring body condition of white rhinos based on the criteria described by Keep (1971).

Index Score	Description
1: Good	Well-muscled pelvic and shoulder area, folk of the flank no prominent, ribs not clearly visible
2: Fair	Deep neck groove, visible spine of the scapula with some atrophy of supra and infra-spinatus muscles
3: Poor	Neck and scapula muscle wasting well advanced, the fold of the flank is very prominent and double
1: Very Poor	Like in “Poor”, marked groove clearly visible along the spine, atrophy of hind legs muscles, clearly visible ribs

**Table 2:** Timeline of key points in the rehabilitation process and release.

May 2018	Arrival of Three White Rhino Females to The Rehab Centre in The Private Game Reserve
June 2018	Adding a sheep as a companion for female C
March 2019	Weaning female C
April 2019	Combining all three females into same enclosure in Rehab Centre
June 2019	Moving animals to the new enclosure in Wildlife Rehabilitation Centre
November 2019	Immobilization, translocation and release of all three animals in the reserve
December 2019	Female C separates form the other two and leads a solitary life

**Table 3:** Points of confirmation of successful release.

Measure of Success	Points in Rehabilitation Care Supporting Success	Confirmation of Success after Releasing
Recovery after immobilization and translocation	Veterinary protocol	Short recovery time, standing position minutes after administering the reversal drugs, walking out of the crates on their own, immediate interest in food
Finding water	Providing animals with access to fresh water	Data from radiocollars: time spent around waterholes
Finding food	Providing access to vegetation consumed by wild rhinos; scattering extra food in different spots in the enclosure	Data from radiocollars: time spent in pasture spots, migrating to different pasture spots
Showing behaviours typical for wild rhinos	Planning the enclosure to resemble the conditions in the wild	Observing behaviours typical for wild rhinos, i.e., mud wallowing, body scratching, horn rubbing
Bond between animals	Keeping 2 older females in one enclosure, sheep company to the younges one, keeping all females in one enclosure fo the last 7 months	2 older females stayed together, the younger one lives solid life
Alert bevaviour	Limiting human-animal contact to minimum	Expressing alert behaviour in case of potential danger, i.e., ear perking, sniffing and monitoring the surroundings
Breeding	No special points	Not confirmed yet
